# Spontaneous Gangrene in Glucosuria

**Published:** 1853-11

**Authors:** 

**Affiliations:** Calvi


*Spontaneous Gangrene in Glucosuria.* By M. Marchal, of Calvi.—Two years ago the author observed a case of spontaneous gangrene in a diabetic patient, who first lost the little toe, and, continuing to void sugar in the urine, eventually succumbed with gangrene of nearly the entire foot. Since this observation, made fifteen years ago, Dr. Landouzy, of Rheims, communicated a case of gangrene of both legs in a diabetic patient. A third case has occurred recently.

M. Marchal was called into consultation, near Paris, to see a patient suffering from two gangrenous spots upon the dorsal region. One, very large, and surrounded by phlegmonous redness and œdema, extended along the outer part of the left thigh. The patient, upon inquiry, stated that, for many years, he had drunk much, and had voided large quantities of urine. The urine, examined by M. Duroy, an experienced chemist, yielded much sugar. As in one of the preceding cases, the patient had suffered frequently from boils in various parts of the body, M. Marchal purposes carrying on his investigations further at a future period, but thought the fact of sufficient importance to lay before the Society.—*Ibid, from L' Union Medicale.*

